# Ruthenium-containing supramolecular nanoparticles based on bipyridine-modified cyclodextrin and adamantyl PEI with DNA condensation properties

**DOI:** 10.1186/s11671-018-2820-y

**Published:** 2018-12-19

**Authors:** Fang Yan, Jian-Shuang Wu, Zhi-Li Liu, Hong-Li Yu, Yong-Hong Wang, Wei-Fen Zhang, De-Jun Ding

**Affiliations:** 1 College of Pharmacy, Weifang Medical University, Weifang, 261053 Shandong China; 2 Collaborative Innovation Center for Target Drug Delivery System, Weifang Medical University, Weifang, 261053 Shandong China

**Keywords:** Supramolecular chemistry, Self-assembly, Ruthenium complexes, Cyclodextrin, Non-viral gene delivery vector

## Abstract

**Abstract:**

Exploring safe and highly efficient gene carriers made from biocompatible constituents has great prospects for clinical gene therapy. Here, a supramolecular gene delivery system was readily constructed by assembling adamantyl-modified polyethylenimine (PEI-Ada) units with a versatile ruthenium bipyridine-modified cyclodextrin (Ru-CD) through host-guest interactions. The photophysical and morphological features of the PEI-Ada@Ru-CD nanoparticles were systematically characterized by techniques including UV-vis absorption spectroscopy, fluorescence spectroscopy, transmission electron microscopy, dynamic light scattering, and zeta potential experiments. The small size and suitably positive zeta potential of the nanoparticles facilitated their cellular uptake and gene transfection. As expected, DNA interaction studies, which were performed using agarose gel electrophoresis and atomic force microscopy, showed that the ability of the nanoparticles to condense DNA was higher than that of the gold standard, i.e., PEI, at low N/P ratios. The design of these ruthenium-containing supramolecular nanoparticles based on bipyridine-modified cyclodextrin and adamantyl PEI has great prospects in the development of gene delivery vehicles.

**Graphical abstract:**

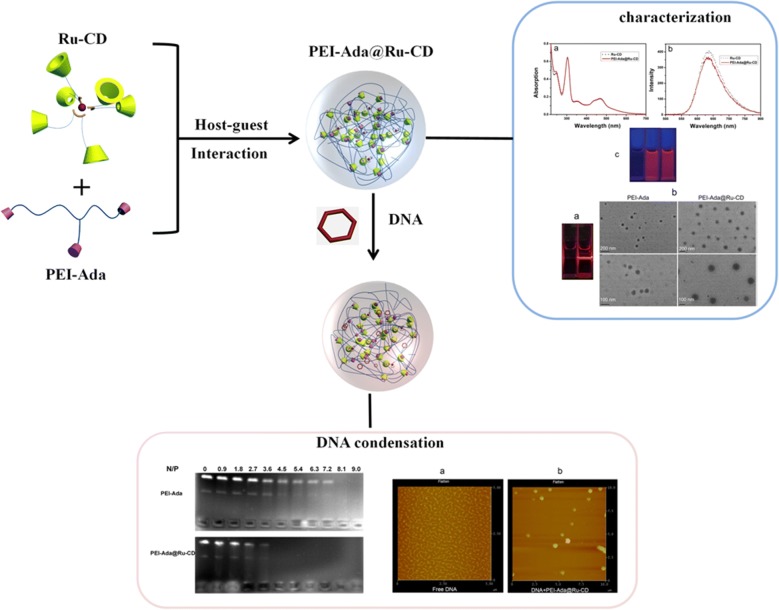

**Electronic supplementary material:**

The online version of this article (10.1186/s11671-018-2820-y) contains supplementary material, which is available to authorized users.

## Introduction

Gene therapy has long been investigated as a promising approach to treat severe diseases [1–3], such as degenerative diseases, cancer, and genetic diseases. This type of therapy aims to cure diseases by introducing genetic material into cells to alter or replace defective genes [3, 4].

Indeed, for successful gene therapy, an efficient delivery system is required. Viral delivery with a high transfection efficiency was the initial impetus for gene therapy, but the safety of viruses in terms of their toxicity, immunogenicity, and quality scale-up production is a concern [5]. As an alternative, synthetic gene vectors, which have many advantages, including low immunogenicity, a desirable DNA loading capacity, and facile manufacturing, have received much attention [6]. To date, a broad range of non-viral systems for genes, including lipids [7, 8], polymers [9, 10], and peptides [11, 12], have been developed. Polyethylenimine (PEI), a commonly used polycation that electrostatically binds and protects DNA, has emerged as a widely studied non-viral gene vector, as reviewed elsewhere [13–15]. However, the clinical application of PEI is severely limited by its toxicity; the ED50 of linear PEI was reported to be 4 mg/kg in BALB/C mice [16]. The toxicity of PEI is possibly due to the binding of intracellular and extracellular components at the positively charged surface [14]. Recent studies indicate that the cytotoxicity could be directly reduced by modifying PEI with carbohydrates [17–19].

Moreover, labelling vectors with organic dyes, quantum dots, carbon dots, or metal complexes has been used for tracking in living systems [20–22]. Particularly, ruthenium complexes are of considerable interest due to their applications in photochemistry and inorganic pharmacology [23, 24]. Ru(II) polypyridyl complexes have emerged as promising novel agents for cell-staining systems due to their intense luminescence, large Stokes shifts, high chemical and photostability, low energy absorption, and relatively long lifetimes [23]. Additionally, because ruthenium complexes are positively charged transition metal complexes, they can efficiently condense DNA, which is also suitable for gene delivery [25, 26]. For instance, Chao et al. provided a new paradigm for developing non-viral gene vectors for tracking DNA delivery based on a dendritic nanosized hexanuclear Ru(II) polypyridyl complex [27]. Bhat et al. reported the use of two new luminescent ruthenium(II) polypyridyl complexes as carriers for DNA delivery [25]. These studies indicate that ruthenium complexes are attractive candidates for DNA carriers and can be used in the design of multifunctional gene delivery systems. However, gene vectors based on ruthenium complexes usually require multiple complicated reactions.

Supramolecular chemistry is known to be a powerful and convenient approach for constructing gene device systems from individually tunable molecular building blocks [28–31]. In particular, the construction of supramolecular self-assembly devices based on cyclodextrins (CDs) and their derivatives for use as gene delivery vectors is an active field because of the natural availability, good water solubility, good biocompatibility, and insignificant toxicity of these materials [28, 32, 33].

**Scheme 1:**
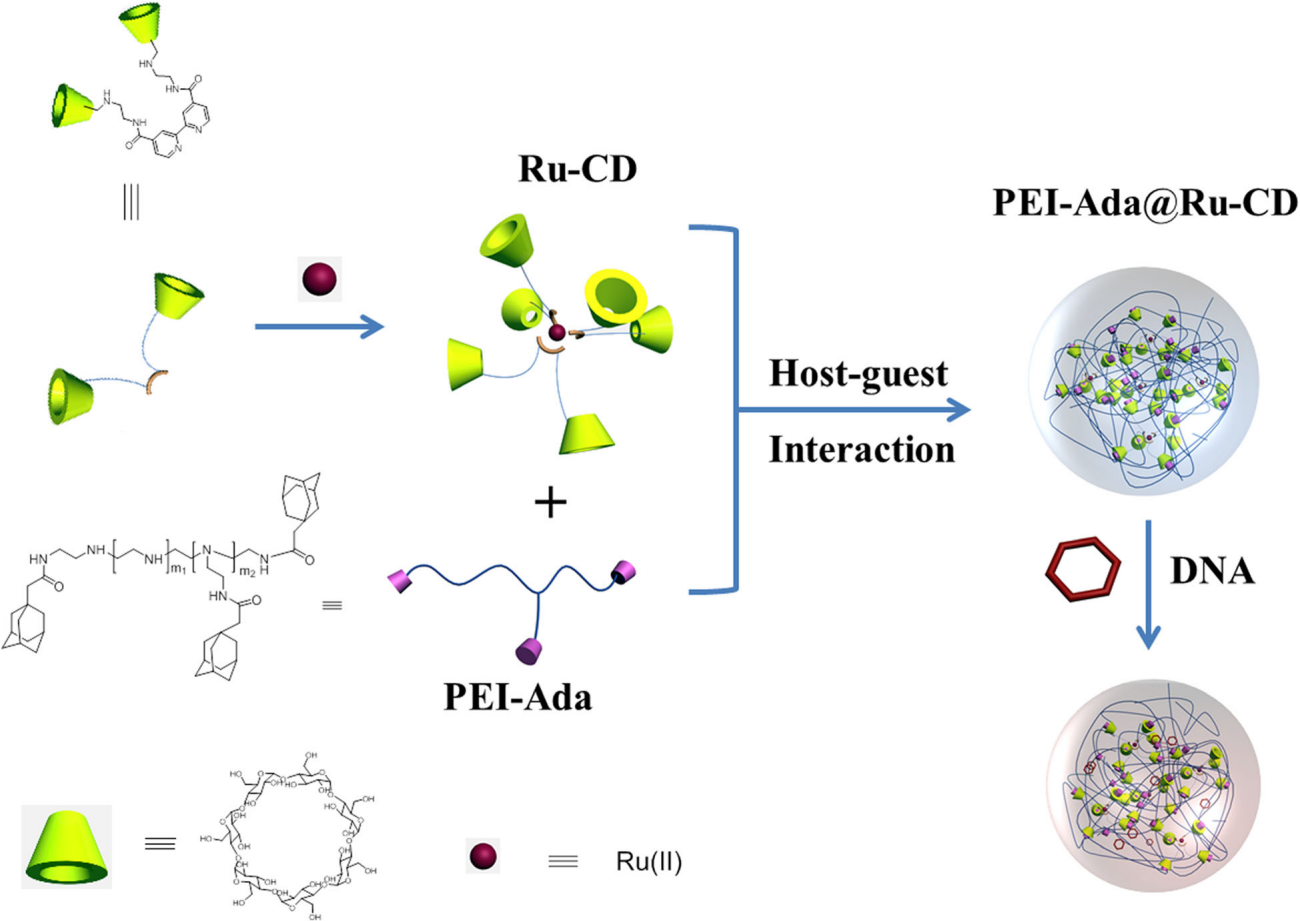
General procedures for the preparation of the ruthenium-containing supramolecular nanoparticles

Inspired by these observations, the host-guest interactions of cyclodextrins were exploited in the design and synthesis of ruthenium-containing supramolecular nanoparticles with DNA condensation abilities as a novel tracking non-viral gene vector (Scheme 1). The inherent advantages for this gene delivery system include the following: (1) ruthenium-containing nanoparticles improve the gene delivery efficiency and provide real-time luminescence imaging for tracking the gene delivery; (2) cyclodextrin, a type of macrocyclic oligosaccharide, is non-toxic, naturally available, stable, and water-soluble, which could improve the solubility of the ruthenium complexes; and (3) this strategy is based on supramolecular self-assembly, avoiding complicated synthesis and separation steps. This work provides promising information for the development of safe, highly efficient non-viral gene carriers and might find wider application in gene therapy.

## Results and discussion

### Synthesis and complexation of PEI-Ada with Ru-CD

**Scheme 2:**
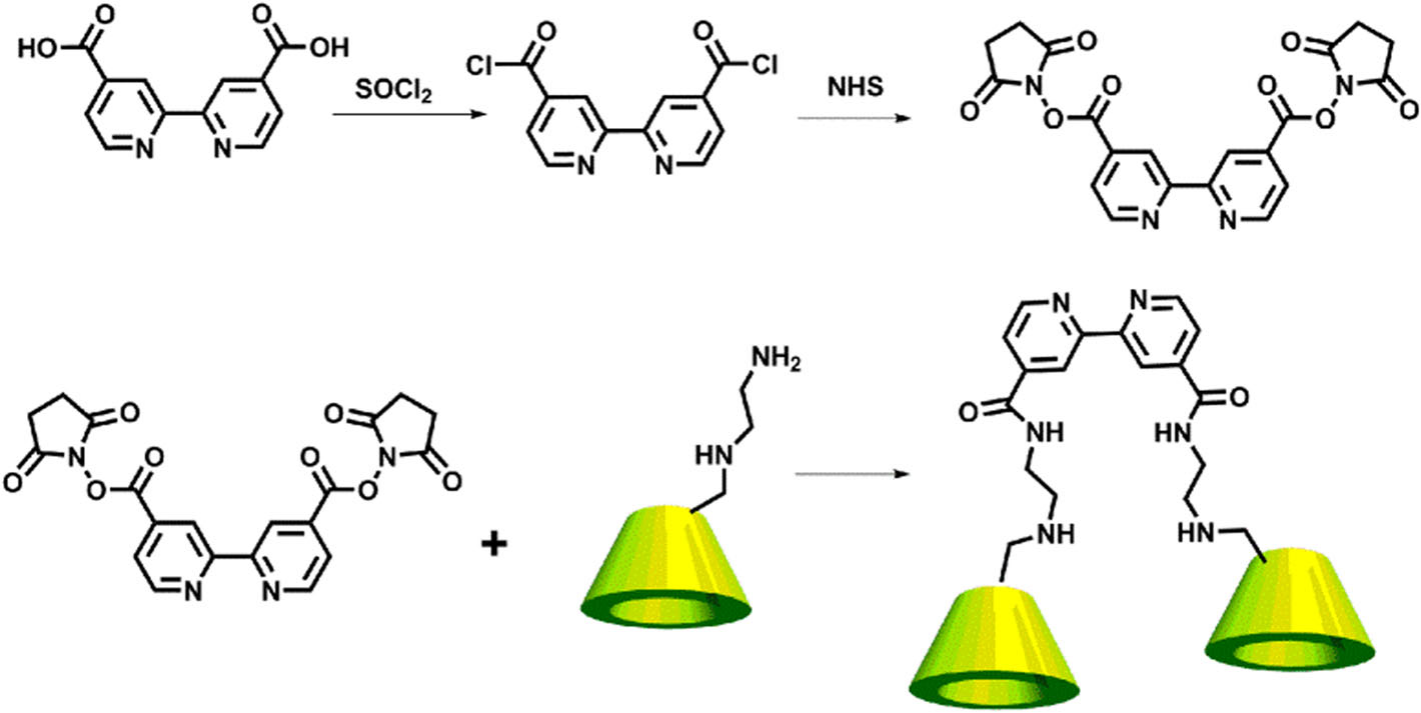
Synthesis route for the bipyridine-modified cyclodextrin

As illustrated in Scheme 2, a β-CD derivative with carboxylated bipyridines (bpy-CD) was synthesized in a moderate yield by a condensation reaction between mono(6-ethylenediamino-6-deoxy)-β-cyclodextrin and 2,2′-bipyridine-4,4′-dicarboxylic acid. Moreover, adamantyl-modified polyethylenimine was synthesized based on a similar method previously reported in the literature [10]. The two complexes had well-defined ^1^H NMR spectra. As shown in Scheme 1, the PEI-Ada@Ru-CD supramolecular system was constructed by the complexation of PEI-Ada with Ru-CD, which involved host-guest interactions between CD and adamantane, in an aqueous solution.

Because the β-CD cavity can strongly bind adamantane derivatives (*K*_*S*_ = 10^4^ M^−1^), the PEI-Ada@Ru-CD supramolecular system could be easily prepared by mixing Ru-CD with PEI-Ada.

### Photophysical properties

**Fig. 1:**
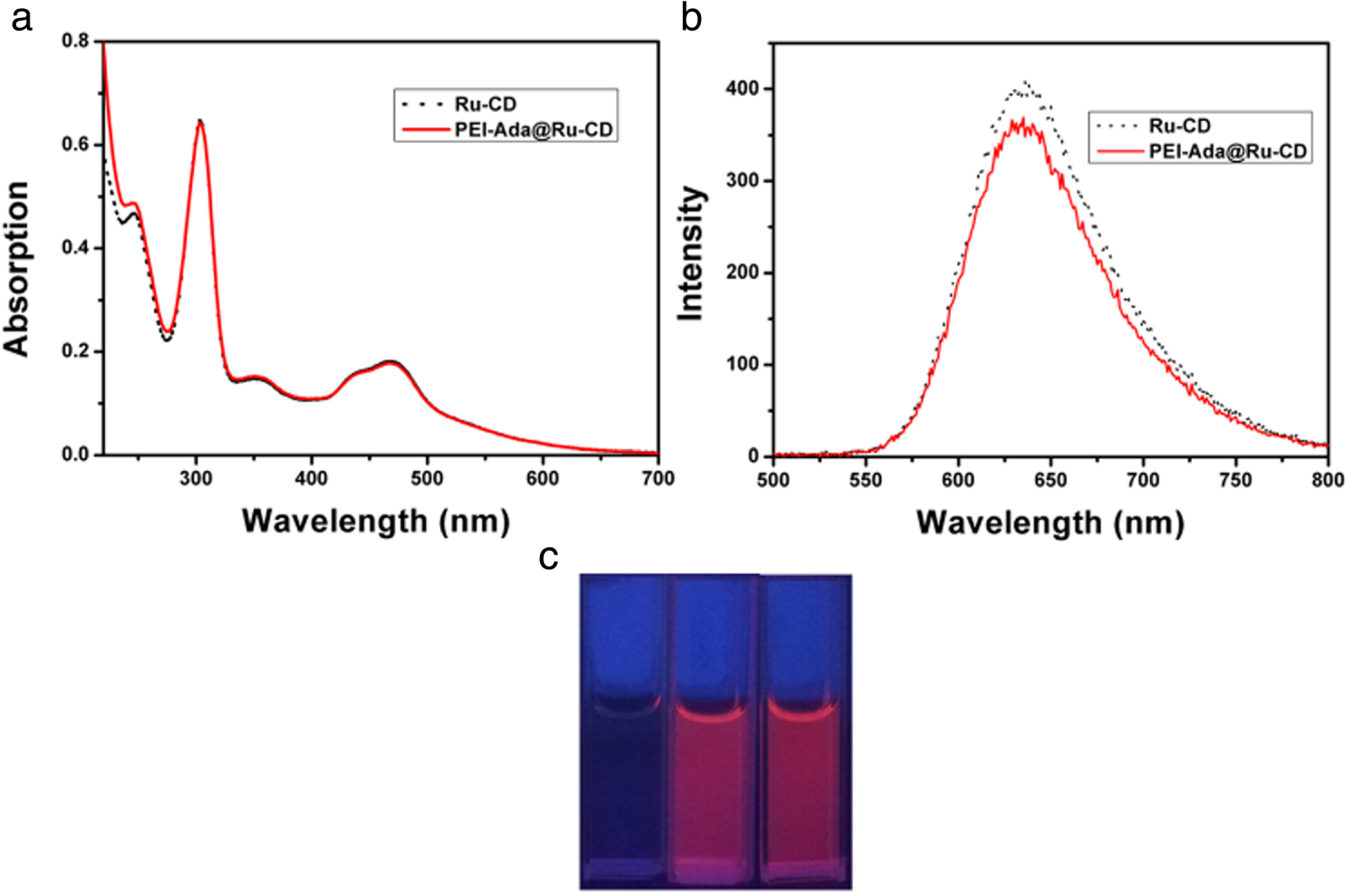
**a** Absorption and **b** emission spectra of Ru-CD and PEI-Ada@Ru-CD in water (10 μM). **c** Luminescence colors of the solutions of PEI-Ada (left), Ru-CD (middle), and PEI-Ada@Ru-CD (right) under a 365 nm lamp

The photophysical properties of the Ru-CD complex and PEI-Ada@Ru-CD nanoparticles in H_2_O are shown in Fig. 1. The ultraviolet–visible spectroscopy (UV-vis) absorption spectra of Ru-CD (Fig. 1a) were characterized by intense absorptions at up to 304 nm in the UV region, which were assigned to the *π*-π* transitions of the aromatic nitrogen donor and polypyridyl ligands, and by three bands with lower intensities associated with metal-to-ligand charge transfer (MLCT) (*dπ*_Ru_-π*_dpb_) transitions in the range of 340 to 550 nm, which is typical for ruthenium(II) polypyridyl complexes [34]. The characteristics of the UV-vis spectra of the PEI-Ada@Ru-CD nanoparticles were similar to those of the Ru-CD complex. Moreover, the Ru-CD complex and PEI-Ada@Ru-CD nanoparticles were highly red luminescent in aqueous solutions (Fig. 1c) under a 365 nm lamp. Excitation of the MLCT band (470 nm) resulted in a broad emission peak centered between 550 and 800 nm (Fig. 1b) [26]. Due to their good fluorescence properties, these nanoparticles have good potential for real-time tracking during delivery and transfection.

### Characterization of the PEI-Ada@Ru-CD nanoparticles

**Fig. 2:**
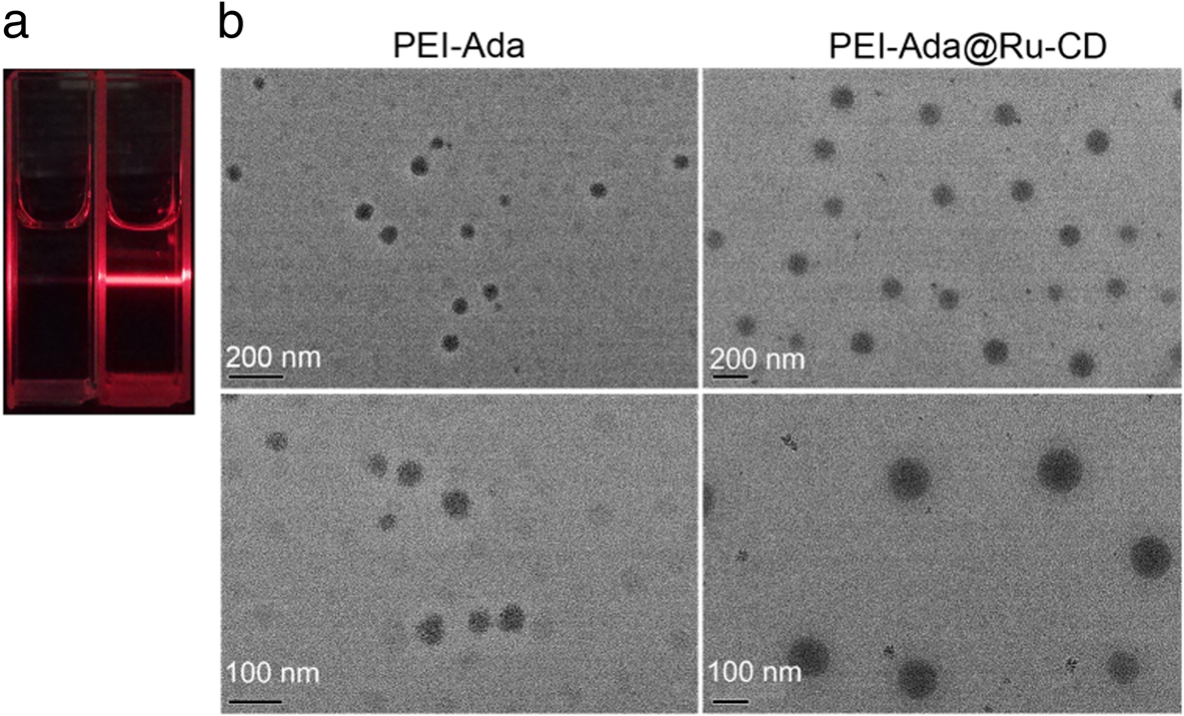
**a** Images of the Tyndall effect of PEI-Ada (left) and the Ru-CD aggregate solution after adding PEI-Ada (right). **b** TEM images of PEI-Ada and PEI-Ada@Ru-CD

To determine the size and structural features of the PEI-Ada@Ru-CD nanoparticles, transmission electron microscopy (TEM), dynamic light scattering (DLS), and zeta potential experiments were performed. First, the Tyndall scattering effect was visible when the solutions were irradiated with a laser, demonstrating their colloidal nature (Fig. 2a). TEM images showing the morphology of PEI-Ada@Ru-CD are presented in Fig. 2b. The PEI-Ada@Ru-CD particles were round and had a homogeneous size distribution with an average diameter of ca. 180 nm. Non-viral gene complexes enter cells via endocytic pathways [35]. Nanoparticles that are smaller than 200 nm have a higher chance of undergoing endocytosis [36]. PEI-Ada also formed spherical nanoparticles of approximately 30 nm. The clearly visible profile in the image in Fig. 2b shows the formation of various highly polymerized supramolecular assemblies.

The size of PEI-Ada@Ru-CD was also measured by DLS. The particle size of PEI-Ada@Ru-CD was determined to be 263 nm with a normal size distribution. In comparison, the PEI-Ada particles had a diameter of ca. 52 nm. Based on these results, it was concluded that the particle hydrodynamic diameter increased as a function of the number of stabilizing layers after the deposition of PEI-Ada on Ru-CD, whereas the size distribution of the PEI-Ada@Ru-CD nanoclusters might have contributed to the lower degree of aggregation [37]. The hydrodynamic diameter of PEI-Ada@Ru-CD measured by DLS was larger than that calculated from the TEM experiments due to the existence of a hydration layer on the outside of the particles in the aqueous solution and to the shrinkage of the drying nanoparticles during the TEM measurements [38].

**Fig. 3:**
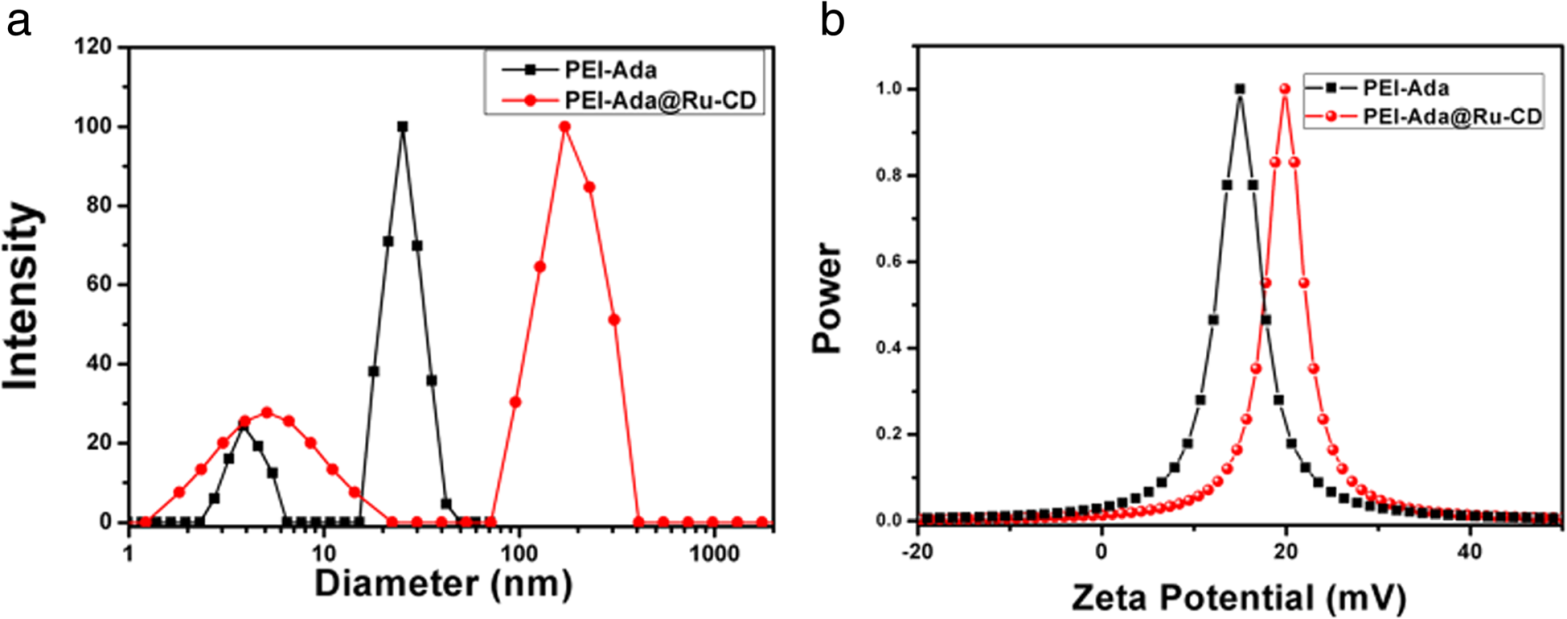
**a** Hydrodynamic diameter distributions and **b** zeta potentials of PEI-Ada and PEI-Ada@Ru-CD

In addition, the zeta potential of PEI-Ada@Ru-CD is another factor that influences its DNA condensation ability and cellular uptake [39]. The PEI-Ada@Ru-CD nanoparticles had a positive zeta potential of + 20 mV (Fig. 3b). Due to the positive charges, the PEI-Ada@Ru-CD complexes should have a good affinity for negatively charged plasmid DNA [28]. In addition, the positive charges make it more likely that these complexes could undergo an adsorptive transcytosis pathway because the endothelial cell external membrane is negatively charged [36].

### DNA condensation ability

**Fig. 4:**
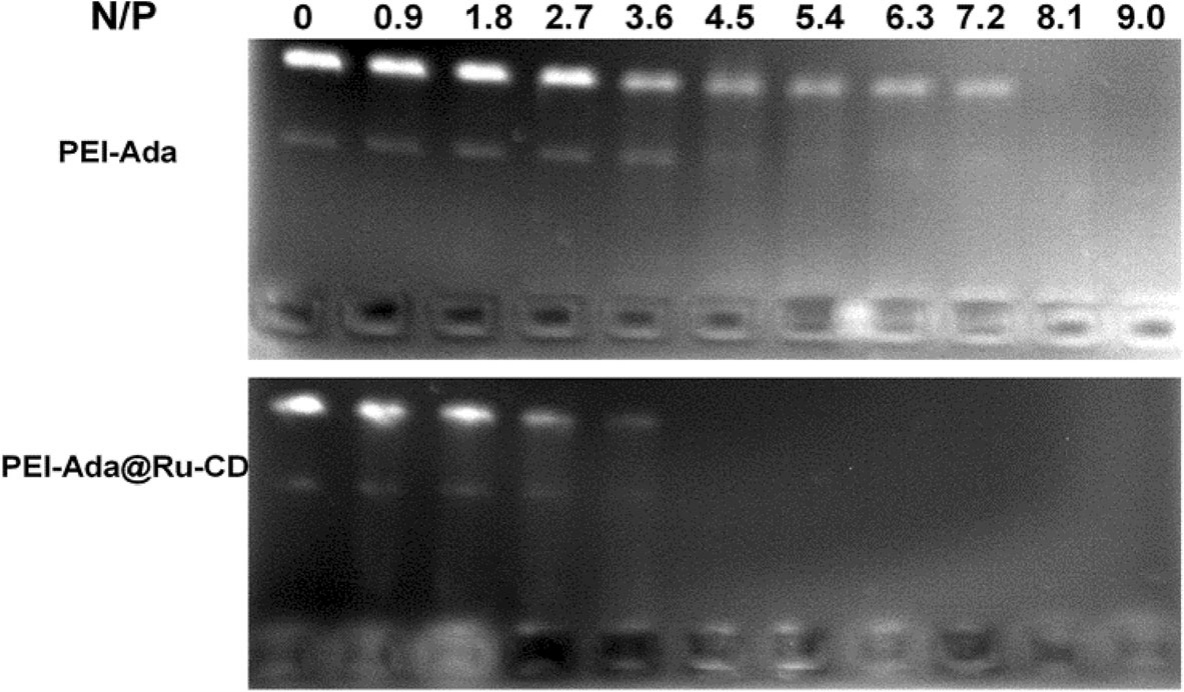
Electrophoretic mobility retardation assay of the pBR322 DNA polyplexes with **a** PEI-Ada and **b** PEI-Ada@Ru-CD at different N/P ratios

Because negatively charged DNA is a non-viral gene carrier, its efficient condensation into nanoparticles through electrostatic interactions is of great importance [28]. The DNA condensation ability of the PEI-Ada@Ru-CD supramolecular nanoparticles was investigated by agarose gel electrophoresis (Fig. 4). A gel retardation assay was performed using circular plasmid pBR322 DNA and the PEI-Ada@Ru-CD nanoparticles or PEI at different N/P ratios on agarose gel. Figure 4 shows that both PEI-Ada and PEI-Ada@Ru-CD could efficiently condense DNA. As the N/P ratio of PEI-Ada@Ru-CD increased, a noticeable DNA retardation was observed in the gel loading wells. The decrease in the electrophoretic mobility of DNA in the presence of the complexes was due to the condensation/aggregation of DNA. Significantly, complete DNA retardation was observed in the presence of PEI-Ada@Ru-CD at N/P ratios of 4.5, 5.4, 6.3, 7.2, 8.1, and 9.0, whereas the corresponding values for the parent PEI-Ada complex were higher (N/P ratio = 8.1, 9.0). These results indicated that introducing Ru-CD enhanced the DNA condensing ability of PEI-Ada, probably because of the supramolecular structure. The formation of amphiphilic gene vectors from the adamantane-β-CD inclusions could improve the density of cationic head groups to improve the DNA condensation ability [28, 33]. Additionally, ruthenium(II) complexes can be used as non-viral gene vectors due to their greater variety of charge states and high DNA binding affinities through the polypyridyl ligands [24, 26].

**Fig. 5:**
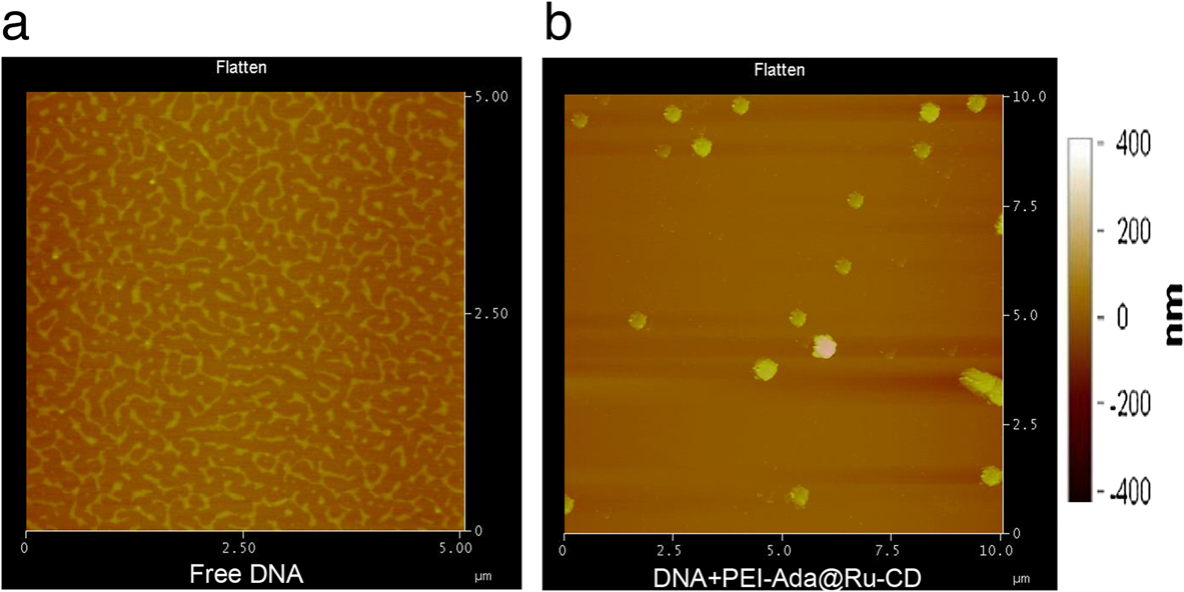
AFM images of **a** free pBR322 DNA and **b** PEI-Ada@Ru-CD with pBR322 DNA at an N/P ratio of 7.2

To elucidate the formation and morphology of the PEI-Ada@Ru-CD/DNA complexes, atomic force microscopy (AFM) was employed. DNA condensates were prepared by incubating a mixture of DNA and the PEI-Ada@Ru-CD nanoparticles (N/P ratio = 7.2) for 15 min at room temperature. As shown in Fig. 5, the unmodified supercoiled pBR322 DNA had a relaxed open loop structure and exhibited little twisting on the mica surface (Fig. 5a). In the presence of the PEI-Ada@Ru-CD supramolecular complex, the originally loose DNA lines became tightly compacted particles with a diameter of ~ 100 nm (Fig. 5b), demonstrating that the nanoparticles efficiently induced DNA condensation. To confirm the stability of the PEI-Ada@Ru-CD/DNA complexes, a DLS particle size analysis was performed (Additional file 1: Fig. S3). The size of the complexes remained nearly unchanged in the presence of serum for 24 h.

### Cell viability and imaging

**Fig. 6:**
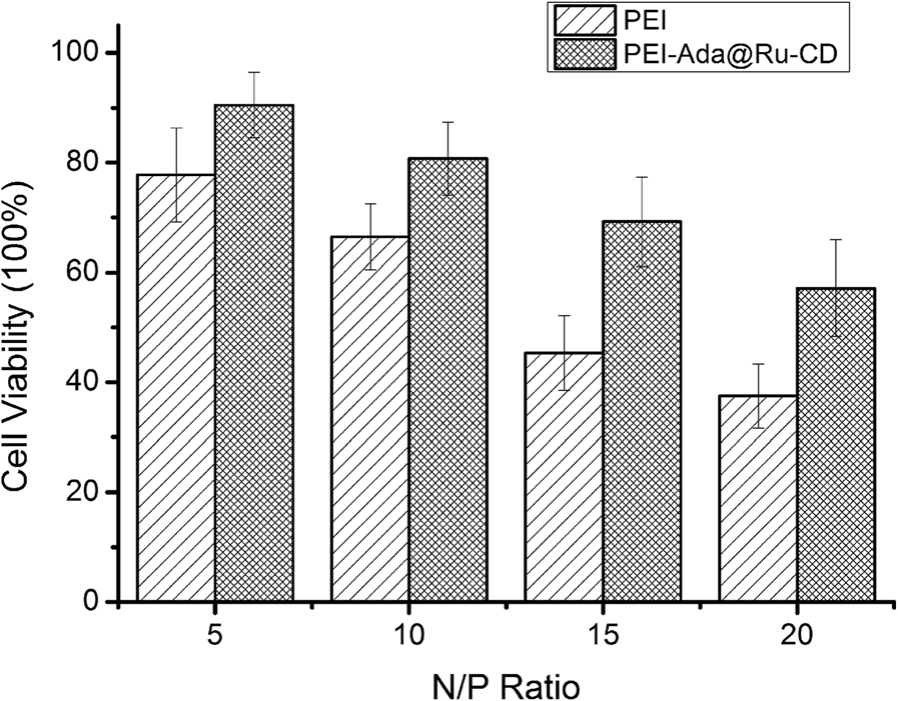
Cell viability of PEI/DNA and PEI-Ada@Ru-CD/DNA complexes at various N/P ratios in A549 cell lines

The biocompatibility of potential gene vectors is one of the major factors that must be considered. To investigate the biocompatibility of the supramolecular gene vector, the viability of A549 cells was examined. As shown in Fig. 6, the cytotoxicity of PEI (25 kDa) and PEI-Ada@Ru-CD increased slightly with increasing N/P ratio, due to the increase in the number of polycations. However, PEI-Ada@Ru-CD exhibited significantly lower cytotoxicity than PEI at all the N/P ratios. These results indicated that PEI-Ada@Ru-CD had better biocompatibility. As reported earlier, the presence of β-CD could shield the deleterious excess charges of PEI, leading to lower cytotoxicity [28, 33].

**Fig. 7:**
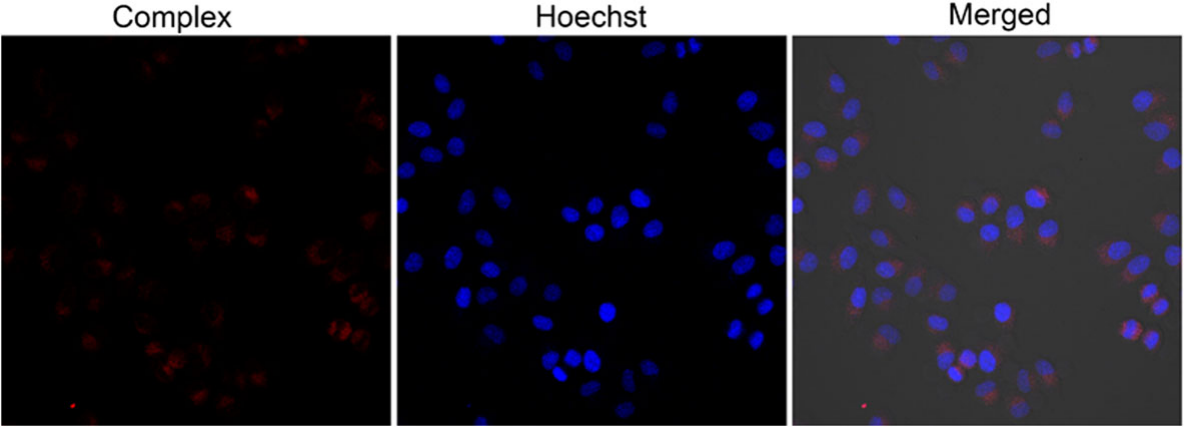
Fluorescent images of A549 cells treated with PEI-Ada@Ru-CD/DNA for 8 h at an N/P ratio of 7.2

Because ruthenium complexes have emerged as promising luminescent tools [24, 25], the cellular uptake of PEI-Ada@Ru-CD was studied with fluorescence microscopy. As shown in Fig. 7, red fluorescence was observed within the cytoplasm, confirming the uptake of the condensates by the cells. A common method for monitoring intracellular uptake is to label viral vectors with organic dyes [20]. However, these substances might alter the gene delivery mechanism and induce side effects. The results of this study demonstrated that the PEI-Ada@Ru-CD system has dual functionalities as a DNA carrier and probe for transport imaging.

Zhuo et al. have developed a supramolecular approach for constructing a versatile gene delivery using β-cyclodextrin and adamantyl-functionalized PEIs [40]. Compared with this study, we constructed a ruthenium-containing supramolecular gene deliver. As cationic groups, the PEI head grafted achieved better transfection activity. And β-cyclodextrin might be useful to improve the bioavailability on gene delivery system. More importantly, the β-cyclodextrin and adamantane offered a powerful and convenient method for fabricating complicated nanostructures. More interesting is that the ruthenium-containing nanoparticle not only improves the gene delivery efficiency, but also is a probe for transport imaging.

## Conclusions

In conclusion, a supramolecular gene delivery system was readily constructed by assembling adamantyl-modified polyethylenimine (PEI-Ada) units with a versatile Ru-CD through host-guest interactions. The photophysical and morphological features of the PEI-Ada@Ru-CD nanoparticles were systematically characterized by UV-vis absorption spectroscopy, fluorescence spectroscopy, TEM, DLS, and zeta potential experiments. As expected, the DNA interaction studies, which were performed using agarose gel electrophoresis and AFM, showed that the DNA condensation ability of the nanoparticles was higher than that of the gold standard, i.e., PEI, at low N/P ratios. This ruthenium-containing supramolecular gene vector has dual functionalities as a DNA carrier and probe for transport imaging, which opens the door for the further development of novel supramolecular gene delivery systems.

### Experiments

#### Materials

All materials and solvents were purchased from commercial suppliers and used as received unless otherwise stated. 4,4-Dimethyl-2,2-bipyridine, RuCl_3_·3H_2_O, dimethyl sulfoxide, branched PEI (MW 25 kDa), β-cyclodextrin, ethidium bromide (EtBr), *N*-hydroxysuccinimide, and supercoiled pBR322 plasmid DNA (stored at − 20 °C) were purchased from Sigma Chemical Company. Reagent grade β-cyclodextrin was crystallized and recrystallized twice with H_2_O and dried in vacuo for 24 h at 368 K. Mono[6-(2-aminoethylamino)-6-deoxy]-β-cyclodextrin [37] and the bipyridine ligands [23] were synthesized according to literature methods. Adamantane-modified polyethylenimine (PEI-Ada) was synthesized according to a previous report [10]. Purified water was obtained from a Milli-Q Plus (Millipore) system and was used in all experimental solutions.

#### Methods and instrumentation

UV-vis spectra were recorded at room temperature using a Thermo 300 spectrophotometer (Thermo Electron Corporation, USA). Fluorescence spectroscopic studies were performed using an F-7000 fluorescence spectrophotometer (Hitachi High-Technologies Co., Ltd., Japan).

^1^H NMR spectra were recorded on a Bruker DMX-400 MHz spectrophotometer with DMSO-d6 as the solvent and SiMe_4_ (TMS) as the internal standard. High-resolution mass spectra were collected on an LC-MS instrument in the ESI mode. For the DLS measurements, the sample solution was filtered through a 0.80-mm filter into a clean scintillation vial and then examined by a laser light-scattering spectrometer (Nano ZS90, Malvern Instruments, UK) equipped with a digital correlator at 636 nm. AFM images were obtained with a Nanoscope IIIa Multimode AFM (Veeco Company, Multimode, Nano IIIa).

#### Synthesis of Ru-CD

A solution of the bipyridine-modified cyclodextrin (300 mg, 0.080 mmol) (synthesized according to the literature method shown in Scheme 2, ESI-MS and ^1^H NMR spectra shown in Additional file 1: Figure. S1–S2) and RuCl_3_ (5 mg, 0.025 mmol) in 6 mL anhydrous *N*,*N*-dimethylformamide was refluxed for 24 h under N_2_. A small amount of H_2_O was added, and the mixture was then poured into acetone (150 mL) to give a reddish-brown precipitate. The crude product was obtained by suction filtration, further purified using a Sephadex G-25 column (mobile phase 0.1 M NH_3_·H_2_O), and then recrystallized from water/acetone (*v*/*v* = 3/1) to give the desired product (220 mg, 65%) as a brown powder: ^1^H NMR (400 MHz, DMSO-d6, ppm) 9.02 (d, 6H), 7.97 (m, 12H), 5.07–5.30 (m, 42H), 2.99–3.96 (m, 276H).

#### Preparation of the PEI-Ada@Ru-CD nanoparticles

Briefly, 0.20 mg of the Ru-CD compound and PEI-Ada (24.65 mg, 1.99 mmol) were added to 5 mL distilled water, and the mixture was ultrasonicated for 5 min. Then, the product was collected by centrifugation and washed with water several times. The final solution was stored at 4 °C and diluted to the desired concentration with deionized water before use.

#### Dynamic light scattering and zeta potential assay

The average hydrodynamic diameter and the zeta potential of the PEI-Ada@Ru-CD nanoparticles were determined by DLS measurements (Nano ZS90, Malvern Instruments, UK). Typically, six runs were performed for each solution, and the average of all the runs was reported.

#### Transmission electron microscopy (TEM)

The samples were prepared by depositing 5 μL of a PEI-Ada@Ru-CD nanoparticle aqueous solution on a carbon film supported on a 300-mesh copper grid and allowing them to air-dry before collecting the images.

#### Atomic force microscopy (AFM)

DNA binding experiments were performed by incubating solutions of the PEI-Ada@Ru-CD nanoparticles with pBR322 DNA (2 ng) for approximately 15 min at room temperature. The samples were placed dropwise on a mica substrate, which was freshly cleaved by removing the top layers with tape. The AFM images were obtained on a Nanoscope IIIa Multimode AFM (Veeco Company, Multimode, Nano IIIa) under ambient conditions.

#### Gel electrophoresis mobility assay

In the gel electrophoresis experiments, negative supercoiled pBR322 DNA was treated with PEI-Ada and PEI-Ada@Ru-CD in 50 mM of a Tris-HCl solution (pH = 7.4), and the mixture was incubated at 37 °C for 30 min. The samples were then analyzed by 1% agarose gel electrophoresis for 1 h at 75 V using a Tris-boric acid-EDTA (TBE) buffer (pH = 8.2) as the running buffer. The gel was stained in a Tris-acetate-EDTA (TAE) buffer containing 5 μg/mL ethidium bromide. The slides were visualized under UV light and photographed for analysis using the Alpha Imager 2200 gel documentation system.

#### Cell viability

The cytotoxicity of PEI-Ada@Ru-CD/DNA complexes was performed in A549 cells using MTT assay as reported [38]. The A549 cells were seeded into 96-well plates (5000 cells/well) and incubated for 24 h. Thereafter, the complexes with N/P ratios ranging from 5 to 20 were added and the cells were incubated for 48 h, then the fresh medium containing the MTT reagent (0.5 mg/mL) for another 4 h. Finally, the medium was replaced with 150 μL DMSO. The microplate reader (Bio-Rad, Model 550, USA) was used to record the absorbance 570 nm.

#### Cellular uptake

The A549 cells were seeded in a 6-well plate. After 24 h, the cell culture medium was changed with 900 μL fresh medium and 100 μL PEI-Ada@Ru-CD/DNA (N/P ratio = 7.2). After being incubated for 8 h, the medium was removed. Subsequently, the cell was fixed with 4% paraformaldehyde before being washed with PBS three times. After that, the Hoechst 33258 was used to stain the nucleus. Then, the cells were imaged using fluorescence microscopy.

## Additional file

Additional file 1: **Figure S1.** ^1^H NMR spectra analysis of bipyridine modified cyclodextrin. **Figure S2.** ESI-MS spectra analysis of bipyridine modified cyclodextrin. **Figure S3.** ^1^H NMR spectra analysis of Ru-CD. **Figure S4.** The stability of PEI-Ada@Ru-CD/DNA nanoparticles (at the N/P ratio of 7.2) in DMEM medium over 24 h. (DOCX 3660 kb)

## Abbreviations

AFM: Atomic force microscopy
bpy-CD: β-CD derivative attached carboxylated bipyridines
DLS: Dynamic light scattering
DMF: *N*,*N*-Dimethylformamide
DMSO: Dimethyl sulfoxide
MLCT: Metal-to-ligand charge transfer (dπ_Ru_-π*_dpb_)
MTT: 3-(4,5-Dimethylthiazol-2-yl)-2,5-diphenyltetrazolium bromide
PBS: Phosphate-buffered saline
PEI: Polyethylenimine
PEI-Ada: Adamantyl-modified polyethylenimine
Ru-CD: Ruthenium bi-pyridine modified cyclodextrin
TBE: Tris-Boric acid-EDTA
TEM: Transmission electron microscopy
TMS: Tetramethylsilane
UV-vis: Ultraviolet–visible spectroscopy
