# Supplementary material for: Ruthenium-containing supramolecular nanoparticles based on bipyridine-modified cyclodextrin and adamantyl PEI with DNA condensation properties
Source: Nanoscale Res Lett. 2018 Dec 19;13:408. doi: 10.1186/s11671-018-2820-y (PMC6300456; doi:10.1186/s11671-018-2820-y)
Supplement: Supplementary file 1 — Figure S1. 1H NMR spectra analysis of bipyridine modified cyclodextrin. Figure S2. ESI-MS spectra analysis of bipyridine modified cyclodextrin. Figure S3. 1H NMR spectra analysis of Ru-CD. Figure S4. The stability of PEI-Ada@Ru-CD/DNA nanoparticles (at the N/P ratio of 7.2) in DMEM medium over 24 h. (DOCX 3660 kb) [file 11671_2018_2820_MOESM1_ESM.docx]

**Additional file**

Ruthenium-containing Supramolecular Nanoparticle Based on Bipyridine Modified Cyclodextrin and Adamantyl PEI with DNA Condensation Properties

Fang Yan^1,2^, Jianshuang Wu^1^_,_ Zhili Liu^1^, Hongli Yu^1^, Yong-Hong Wang^1^, Weifen Zhang^1,2*^, Dejun Ding^1,2*^

^1^College of Pharmacy, Weifang Medical University, Weifang 261053, Shandong, China

^2^Collaborative Innovation Center for Target Drug Delivery System, Weifang Medical University, Weifang 261053, Shandong, China


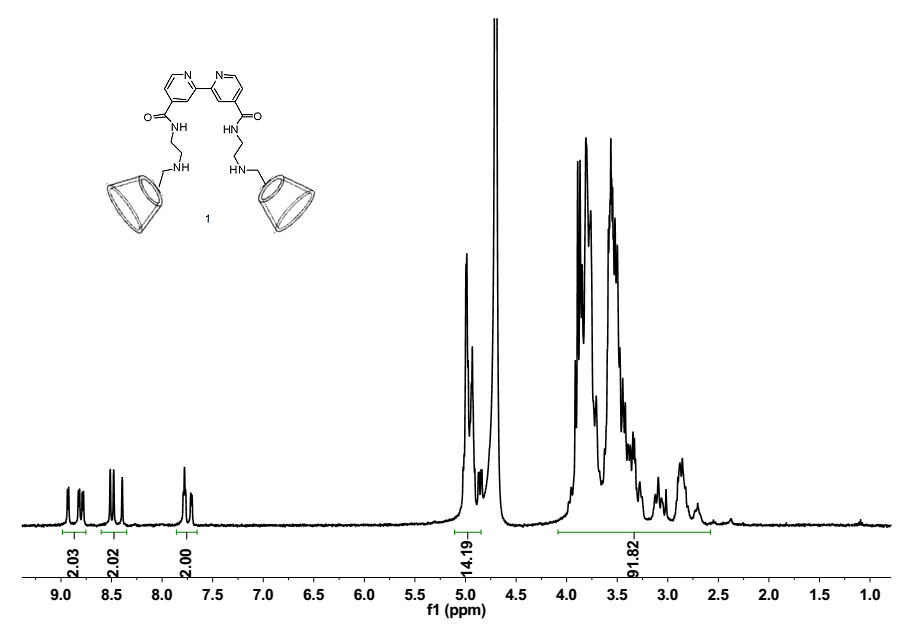


Figure S1. ^1^H NMR spectra analysis of bipyridine modified cyclodextrin.


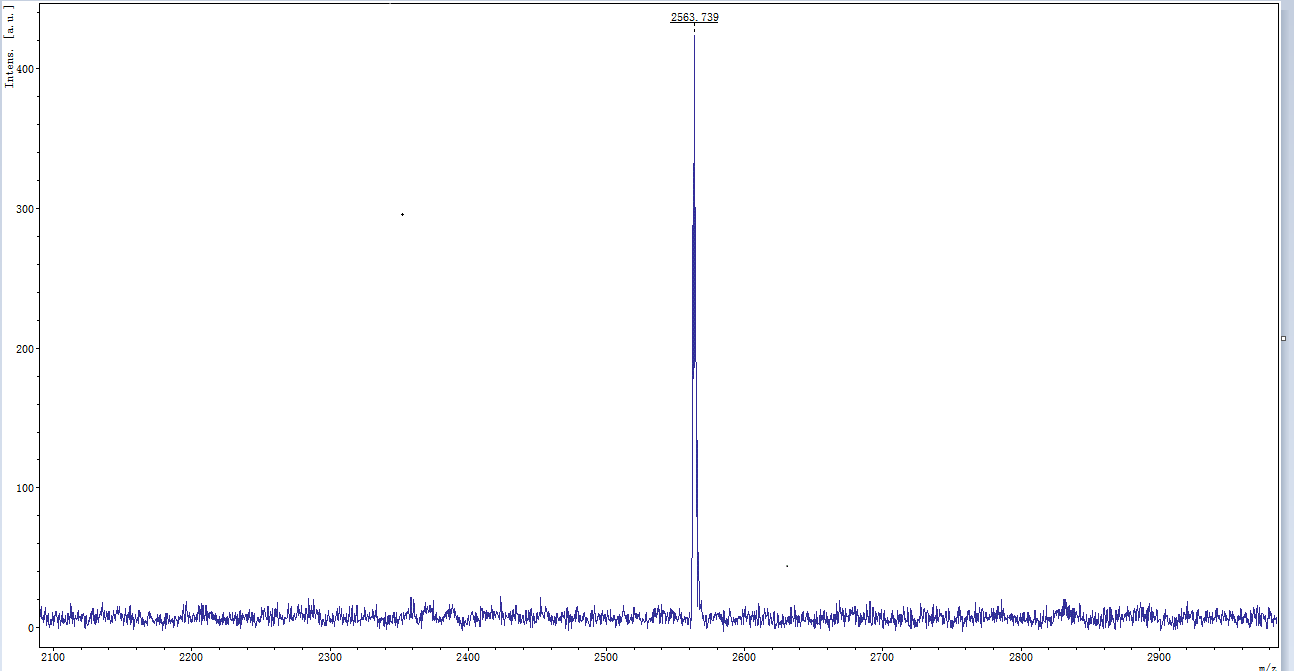


Figure S2. ESI-MS spectra analysis of bipyridine modified cyclodextrin.


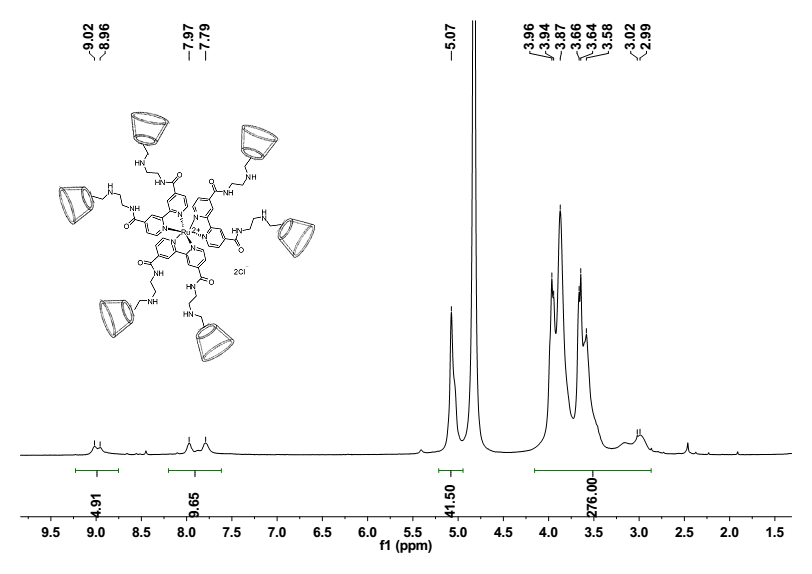


Figure S3. ^1^H NMR spectra analysis of Ru-CD.


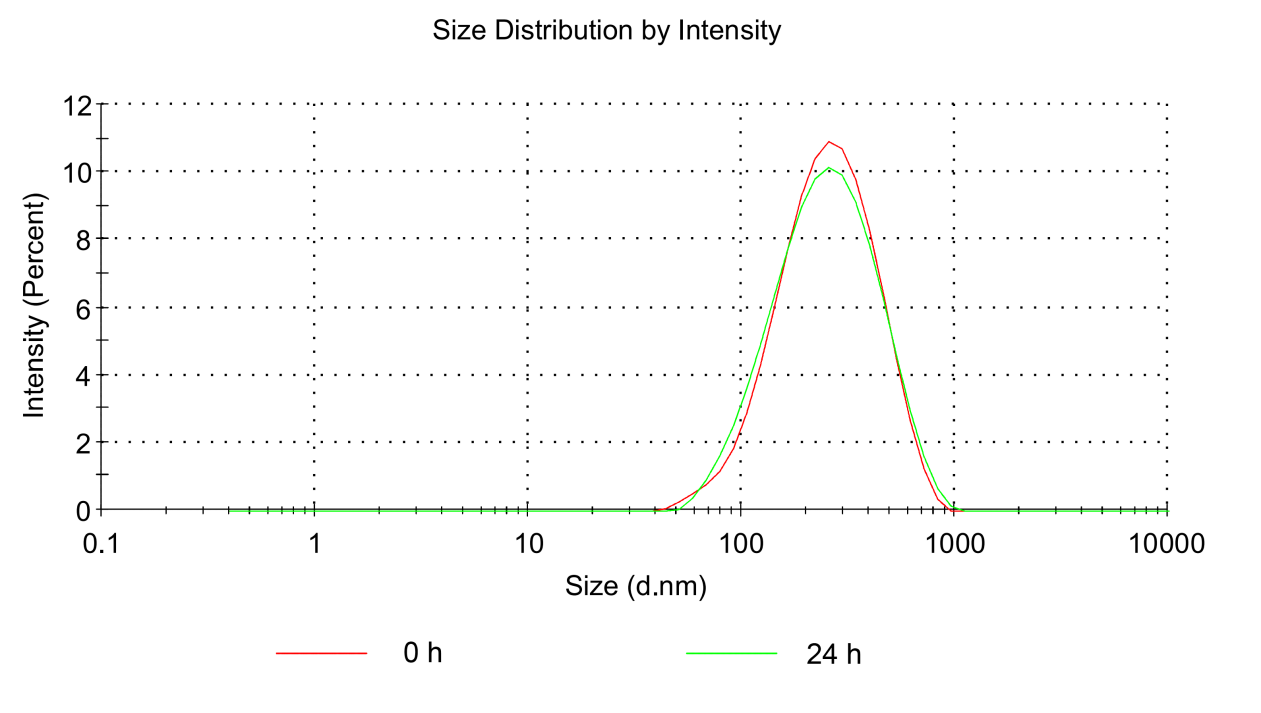


Figure S4. The stability of PEI-Ada@Ru-CD/DNA nanoparticles ( at the N/P ratio of 7.2) in DMEM medium over 24 h.
